# Supplementary figures and images for: Vegetation characteristics control local sediment and nutrient retention on but not underneath vegetation in floodplain meadows
Source: PLoS One. 2021 Dec 2;16(12):e0252694. doi: 10.1371/journal.pone.0252694 (PMC8638890; doi:10.1371/journal.pone.0252694)

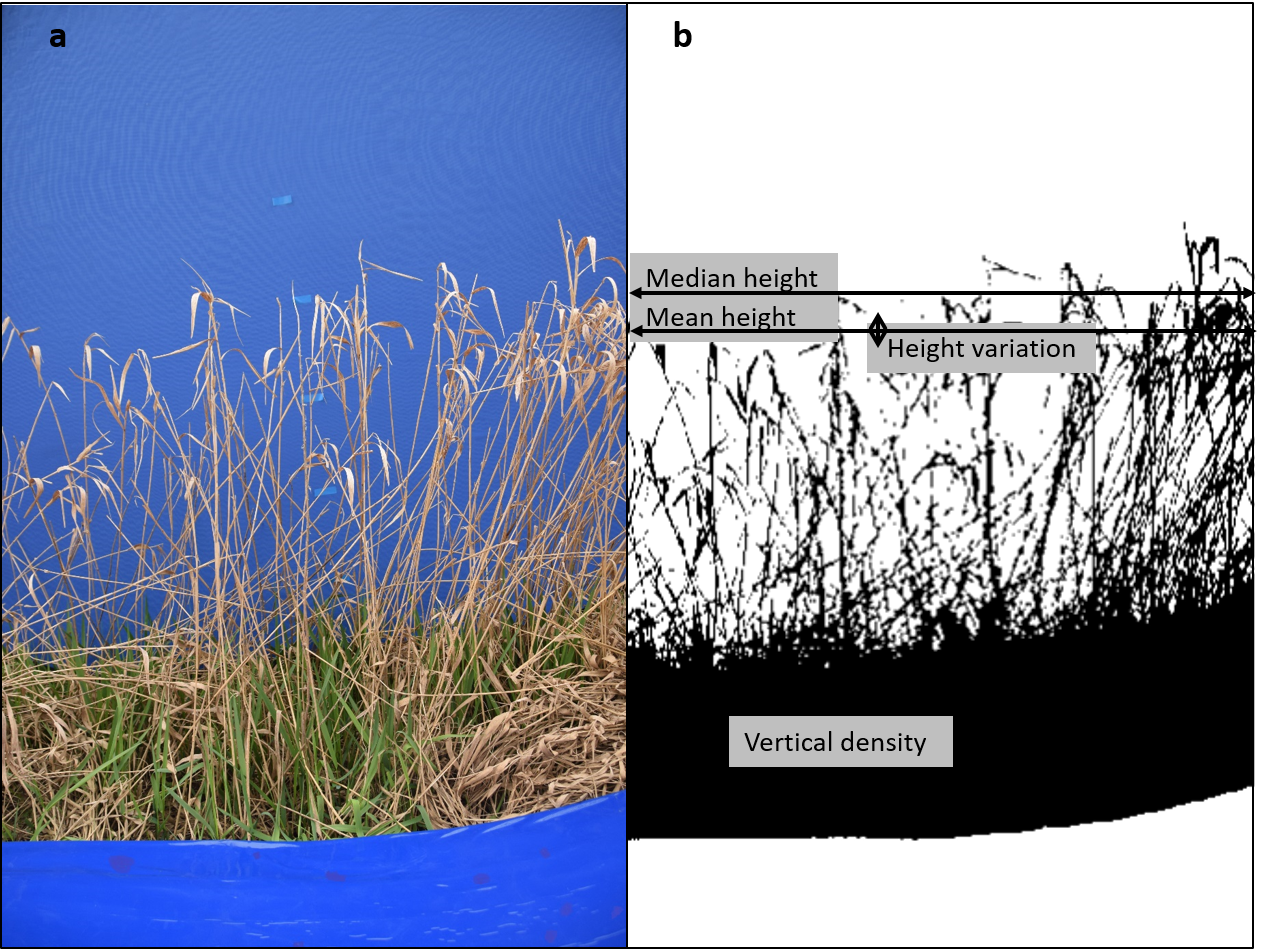

Supplement: S1 Fig — a) Original image with blue background wall and blue flooring material in front. b) Automatically analyzed images for the following predictors as explained in Table 1: Vertical density = percent of vegetation pixels on the image of standard size, Mean height = mean height of vegetation pixels on the image, Median height = median height of vegetation pixels on the image, and Height variation = standard deviation of vegetation pixel height on the image. (TIF) [file pone.0252694.s001.tif]

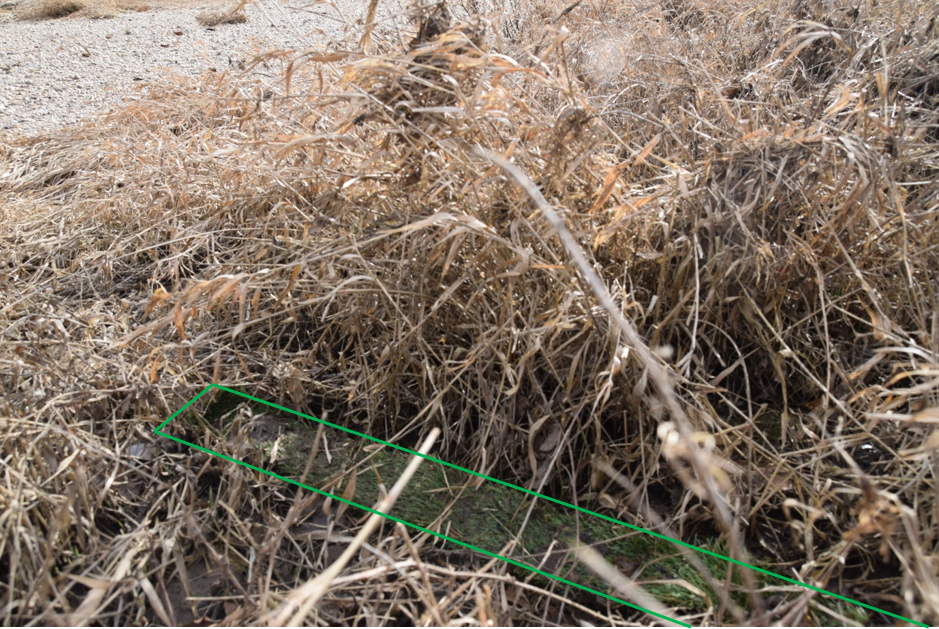

Supplement: S2 Fig — Picture of a sediment trap in the field. (TIF) [file pone.0252694.s002.tif]

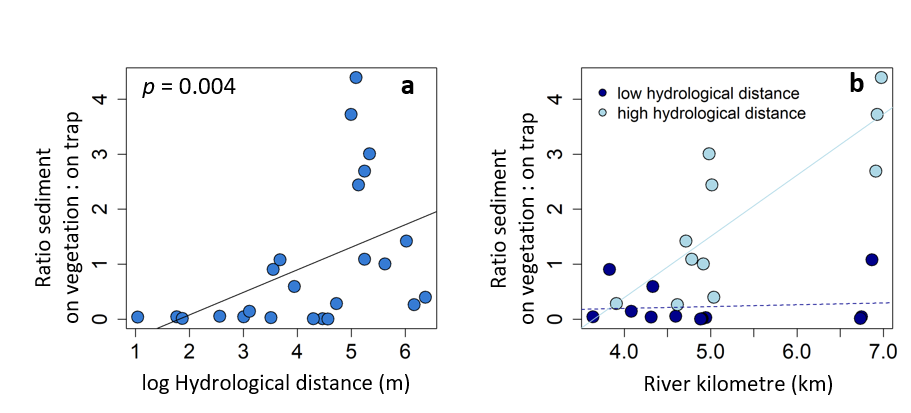

Supplement: S3 Fig — Ratio of sediment on vegetation to sediment on traps. (TIF) [file pone.0252694.s003.tif]
