# Supplementary material for: Vegetation characteristics control local sediment and nutrient retention on but not underneath vegetation in floodplain meadows
Source: PLoS One. 2021 Dec 2;16(12):e0252694. doi: 10.1371/journal.pone.0252694 (PMC8638890; doi:10.1371/journal.pone.0252694)
Supplement: S1 Table — Statistical model results of the ratio sediment on the vegetation to sediment on the traps. (PDF) [file pone.0252694.s004.pdf]

Ratio sediment on vegetation : on traps					
	**Estimate**	**Std. Error**	**t value**	**Pr(>|t|)**	**Sig**
**(Intercept)**	0.988	0.164	6.010	7.09E-06	***
**River kilometre**	0.853	0.176	4.861	9.46E-05	***
**log Hydrological distance**	0.547	0.168	3.255	0.004	**
**River kilometre : log Hydrological distance**	0.619	0.180	3.440	0.003	**
