# Supplementary material for: Vegetation characteristics control local sediment and nutrient retention on but not underneath vegetation in floodplain meadows
Source: PLoS One. 2021 Dec 2;16(12):e0252694. doi: 10.1371/journal.pone.0252694 (PMC8638890; doi:10.1371/journal.pone.0252694)
Supplement: S2 Table — Statistical model results of carbon, nitrogen and phosphorus on the vegetation and on the traps. (PDF) [file pone.0252694.s005.pdf]

Carbon on vegetation

	Estimate	Std. Error	t value	Pr(>|t|)	Sig
**(Intercept)**	0.014	0.775	0.018	0.986	
**Sediment on vegetation**	0.125	0.018	6.757	2.48E-06	***
**Log Hydrological distance**	2.074	0.507	4.094	0.001	***
**Biomass**	0.899	0.522	1.723	0.102	
**Highest flower 16**	0.966	0.508	1.901	0.073	
**Leaf area**	-0.828	0.418	-1.982	0.063	

Nitrogen on vegetation

	Estimate	Std. Error	t value	Pr(>|t|)	Sig
**(Intercept)**	0.154	0.042	3.644	0.002	**
**Sediment on vegetation**	0.006	0.001	5.940	1.02E-05	***
**Log Hydrological distance**	0.074	0.022	3.412	0.003	**
**Elevation above river**	-0.042	0.021	-2.010	0.059	.
**Biomass**	0.079	0.025	3.114	0.006	**

Phosphorus on vegetation

	Estimate	Std. Error	t value	Pr(>|t|)	Sig
**(Intercept)**	0.099	0.004	26.258	8.38E-16	***
**Sediment on vegetation**	0.061	0.005	12.676	2.08E-10	***
**Log Hydrological distance**	0.014	0.004	3.312	0.004	**
**Elevation above river**	-0.005	0.004	-1.297	0.211	
**Biomass**	0.008	0.005	1.743	0.098	.
**Leaf pubescence**	0.005	0.004	1.272	0.219	

Carbon on trap

	Estimate	Std. Error	t value	Pr(>|t|)	Sig
**(Intercept)**	12.089	9.445	1.280	0.215	
**Log Sediment on trap**	36.449	5.560	6.555	2.18E-06	***
**River kilometre**	17.163	6.836	2.511	0.021	*
**Precipitation**	24.009	13.480	1.781	0.090	.

Nitrogen on trap

	Estimate	Std. Error	t value	Pr(>|t|)	Sig
**(Intercept)**	0.774	0.694	1.116	0.278	
**Log Sediment on trap**	2.584	0.409	6.325	3.57E-06	***
**River kilometre**	1.239	0.502	2.467	0.023	*
**Precipitation**	1.903	0.990	1.922	0.069	.

Phosphorus on trap

	Estimate	Std. Error	t value	Pr(>|t|)	Sig
**(Intercept)**	0.668	0.180	3.718	0.002	**
**Log Sediment on trap**	1.081	0.111	9.700	2.41E-08	***
**River kilometre**	0.254	0.130	1.957	0.067	.
**Highest flower 16**	-0.169	0.111	-1.528	0.145	
**Precipitation**	0.346	0.260	1.328	0.202	
