# Supplementary material for: Vegetation characteristics control local sediment and nutrient retention on but not underneath vegetation in floodplain meadows
Source: PLoS One. 2021 Dec 2;16(12):e0252694. doi: 10.1371/journal.pone.0252694 (PMC8638890; doi:10.1371/journal.pone.0252694)
Supplement: S1 File — (DOCX) [file pone.0252694.s006.docx]

Dear Editor,

Please find enclosed a manuscript titled "Vegetation characteristics control sediment and nutrient retention on but not underneath vegetation in floodplain meadows" for consideration for publication in *PLoS ONE*.

We thank you for the positive assessment of our work. In addition, we thank you and the reviewers for the critical and mindful reviews and comments, it clearly helped to improve the manuscript a lot. We addressed every comment in the document “Response to Reviewers” and made the changes visible in the document “Revised Manuscript with Track Changes”.

We would like to extend our Funding Statement by another sentence, as a whole it should state the following:
“C.W. received funding from the German Federal Ministry of Education and Research (BMBF) and the German Federal Agency for Nature Conservation (BfN) for the project: Wilde Mulde - Revitalization of a wild river landscape in Central Germany (Funding label: 01LC1322E). There was no additional external funding received for this study.”

Further we would like to extend the Data Availability Statement with the DOI of the data set: Data are available from the Data Repository iData (https://doi.org/10.25829/idiv.3479-10-1609).

Finally, we would be delighted if you would consider our resubmission for publication in *PLoS ONE*. If you should require anything further regarding this submission, please do not hesitate to contact us.

Yours sincerely,

Lena Kretz (on behalf of all authors)
