# Supplementary material for: Vegetation characteristics control local sediment and nutrient retention on but not underneath vegetation in floodplain meadows
Source: PLoS One. 2021 Dec 2;16(12):e0252694. doi: 10.1371/journal.pone.0252694 (PMC8638890; doi:10.1371/journal.pone.0252694)
Supplement: S2 File — (DOCX) [file pone.0252694.s007.docx]

Journal requirements, editor comments, reviewer comments

Response by the authors (line numbers in the “Revised Manuscript with Track Changes”)

1. Please ensure that your manuscript meets PLOS ONE's style requirements, including those for file naming. The PLOS ONE style templates can be found at

We rechecked the style requirements for PLOS ONE also about file naming.

2.  Thank you for stating in your Funding Statement:

We included the further statement into the Funding Statement and mentioned it in the cover letter.

“C.W. received funding from the German Federal Ministry of Education and Research (BMBF) and the German Federal Agency for Nature Conservation (BfN) for the project: Wilde Mulde - Revitalization of a wild river landscape in Central Germany (Funding label: 01LC1322E). There was no additional external funding received for this study.”

3. We note that you have stated that you will provide repository information for your data at acceptance. Should your manuscript be accepted for publication, we will hold it until you provide the relevant accession numbers or DOIs necessary to access your data. If you wish to make changes to your Data Availability statement, please describe these changes in your cover letter and we will update your Data Availability statement to reflect the information you provide.

The data are preliminary accessible: https://doi.org/10.25829/idiv.3479-10-1609

4. Please include your full ethics statement in the ‘Methods’ section of your manuscript file. In your statement, please include the full name of the IRB or ethics committee who approved or waived your study, as well as whether or not you obtained informed written or verbal consent. If consent was waived for your study, please include this information in your statement as well.

To our knowledge there is no further ethics statement needed for our study.

Additional Editor Comments (if provided):

Overall this is an interesting empirical dataset which provide a wider understanding of dynamics of nutrients and sediments in a river floodplain. The paper is logically presented and well written, but both reviewers have made a range of comments regarding the overall context with which the research is framed. Reviewer 1 has made several comments which require significant explanation and clarification. They suggest that the research needs to framed within existing literature related to key concepts of floodplain sedimentation more completely. Similarly, reviewer 2 has made comments that they would like to see the paper reflect on hydrogeomorphological context more overtly. Both reviewers require more details to explain and justify the overall study design and methods as well. Finally, limitations of research need to be presented and explained more clearly. Clearly given the range of issues raised by both reviewers this work will require major revision before it would be acceptable for publication.

Dear editor, thanks for the opportunity to revise our manuscript.

We carefully revised the above-mentioned aspects, by including more references with an explicit focus on the concepts of floodplain sedimentation by hydrogeomorphological factors related to the flood event itself. Therefore, we considered the suggested paragraph of reviewer 2 and added references and a few additional aspects. We put quite some effort on the literature research, however we cannot preclude that we missed any work of high importance. If this is the case, we would be grateful, if reviewer 1 would be so kind to indicate which specific publications are missing in her/his view. We further improved the methods section with more comprehensive information on the study design and supporting figures, as well as by including more details about the flood event itself in the methods and the discussion section. We also discuss the limitations of the study in an additional outlook section at the end of the discussion section and in the conclusion.

Reviewers' comments:

Reviewer #1:

The character of sediment that accumulated on the River Mulde floodplain during a flood event is reported in this manuscript. The main focus of the manuscript is to unpack the role of floodplain vegetation on influencing the amount of sediment and associated nutrient concentrations that accumulated on the vegetation and directly onto the floodplain surface underneath the vegetation. Floodplain ecosystem processes and their role in determining the source – sink behaviour of floodplains is important for river science. Thus the topic of this manuscript is relevant to ecosystem ecologists and river scientists. I cannot recommend this manuscript for publication, and are between the need for a major revision or rejection. This recommendation is based on the following.

• Limited acknowledgement of the vast literature on the subject of floodplain sedimentation and the role of vegetation and surface complexity as roughness elements in the in process of sediment accumulation. Key concepts of floodplain sedimentation and supporting references are missing. Thus the narrative of floodplain sedimentation in the manuscript is limited, resulting in the advance of the subject matter being limited.

We kindly ask the reviewer to clarify on specific literature on the subject. To respond to the criticism we did an extensive additional literature review, but for further improving our manuscript we would be grateful for concrete hints on which papers of particular importance or even grey literature such as agency reports we might have missed.

Nevertheless, we now extended our reference list and also added two new paragraphs in the introduction, as suggested by reviewer 2, where we provide further information and references as you can read in the following:

“Furthermore, models predict that the inundation depth increase the floodplain roughness and with that might increase sedimentation on the floodplain (12).” (line 61-63)

“Flood magnitude and duration are the fundamental controls on the delivery of sediment transported by a river from its channel onto its floodplain (45). Flood magnitude and duration also affect the spatio-temporal distribution of depth and velocity of the inundating water as it flows across the floodplain surface (46).” (line 141-144)

“First, floodplain topography imposes strong local variations on the depth and velocity of the flood water (20,21,44). Second, any vegetation cover on the floodplain surface interacts with the flood water, slowing local flow velocities and thus encouraging the settlement of sediment particles from the water (20,21,44). However, because the structure of herbaceous vegetation is flexible, it can respond to high flow velocities by bending and adopting a more streamlined form, and this leads to changes in its ability to intercept and retain sediment as flow depth and velocity vary through time and across space (47–49).” (line 146-153)

“In the present case, we investigated a flood of relatively low magnitude that occur on average biannually, but others already emphasised the importance of low magnitude, but higher frequent floods for nutrient retention on floodplains (50)”. (line 166-169)

• Conceptual weakness. Rates of floodplain sedimentation and the character of sediment accumulating on various floodplain surfaces during flooding are influenced by many factors operating at a range of scales. There is no appreciation of the importance of scale and its implications in this study. For example, rates of floodplain sedimentation, irrespective of floodplain surface condition (i.e. types of vegetation and or variable topographic roughness) have previously been shown to be controlled by sediment supply factors and or the dynamics of sediment movement during flood events. While others show the importance of vegetation roughness at a point as the driver on floodplain sedimentation. This study focuses solely on the influence of a narrow range of vegetation factors thereby ignoring important multi-factorial influences – it is scale limited in a process sense. As a result the study does not add to the body of knowledge on floodplain sedimentation.

We included “local” in the title, to be more specific about the scale. “Vegetation characteristics control local sediment and nutrient retention on but not underneath vegetation in floodplain meadows” (line 1-4).

Our study indeed focuses on the vegetation aspect of sediment retention on floodplains, and not on sediment reply or larger scale factors. While we agree with the reviewer that this means that our study has - like most study - a limited scope (as we acknowledge in line 70-72 and 487-491), it is impossible to study all factors driving sedimentation simultaneously. We respectfully disagree with the reviewer that this means that our study does not add any knowledge. Quite to the contrary, other studies have also shown that vegetation can influence sediment retention (18, 19), although these earlier studies did not disentangle the influence of different structural features of the vegetation nor did they investigate the differences of on-plant versus underneath the vegetation sedimentation, as we did in our study. These results complement and add to our current understanding considering different traits of floodplain vegetation. To address the points raised, we also added some more abiotic and flood related aspects in a separated paragraph of the introduction to clarify that the set of drivers and their interactions (line 141-159).

We added “the flood” as a factor that influence sediment retention in (line 76-77). “Sediment retention is a complex phenomenon that depends on the flood and different biogeomorphic processes in the floodplain (18, 19).”

In addition, we clarified the scale we worked at in the study aim. “Thus, the aim of this study was to analyse combined effects of vegetation characteristics and selected topographic features at the scale of vegetation patches on the floodplain.” (line 172-174)

• Study design and methods. Three key points of concern here:

1). The study design used determined sedimentation on the vegetation and directly underneath the vegetation. There was no control site without the influence of vegetation. The influence of vegetation on local surface sedimentation (ie. the high degree of interactions) confounds the study design.

The focus of the study was to analyse the structural characteristics of the vegetation that explain the variation of sediment retention underneath and on the vegetation. Other studies (references 18-22, cited in line 76-80) already found that vegetation in general plays an important role to capture sediment on floodplain; so, to clarify: the aim of our study was not to demonstrate that vegetated floodplains differ in sedimentation from floodplains without vegetation, but to show how different vegetation compositions and structures affect sedimentation.

Moreover, there is no attempt to quantify either the roughness or trapping ability of the floodplain vegetation or the floodplain surface roughness or complexity; both of which are significant parameters in trapping sediment on floodplains. There is vast literature on both aspects as well as routine ways to calculate.

We are a bit uncertain in which direction the comments argues. The “trapping abilities of the floodplain vegetation” we directly quantified as the structural characteristics of the vegetation, such as biomass, mean height and lateral density.

As far as we know, there are model approaches for sediment retention on floodplain available, however these model approaches, if at all, include the vegetation as an idealized element or at a very rough scale (forest vs meadow), however not at the scale on which we investigated the effects of the vegetation structure (Manners et al. 2013, Camporeale et al. 2013, Natho & Venohr 2014, James 2018). We added a sentence about this in the outlook section.

“Our findings will help at the small scale to improve existing model approaches to predict sediment and nutrient retention on floodplains (12,20,50,82).” (line 557-558)

2). Nutrient analysis was performed on bulk sediment less than 2mm in size. Nutrient concentrations, especially for P and C do vary markedly between individual particle size intervals, with particles <0.063mm having naturally elevated concentrations. Any variation in nutrient concentrations could simply reflect variations in particle size.

It is true that variation in nutrient concentration might simply reflect variation in particle size, however this is what we emphasize in the discussion (“Thus, we can derive that the vegetation…” line 514-516). The particle size might cause the nutrient retention, but the key result is that we find more nutrients further away from the river, where there is less sediment retention.

We also added a further reference in line 516: River & Richardson 2018 (80).

3). Statistical analysis of the data. A series of single regression analyses were performed. This is a very rudimentary treatment of an interesting multifactorial data sets that does not fully explore the character of floodplain sedimentation. Multi variate analyses or stepwise regression would add to the analyses of the data.

Actually, we did not perform single regression analyses. As we explained in our original MS: “We used the candidate variables listed in Table 1 as explanatory variables; in the ratio model we additionally included the interaction of the river kilometre and the hydrological connectivity, while in the C, N and P models, we additionally used the sediment amount as an explanatory variable. To meet model requirements regarding the normality of the error distribution, the two variables, “sedimentation on traps” (except for the ratio of sediment on vegetation to on traps) and “hydrological distance”, were natural log-transformed. We scaled all continuous variables to ensure comparability of the model estimates. To avoid multicollinearity, we removed explanatory variables with a variation inflation factor above 5.0 (vif function, car library, 66). With the remaining variables, we ran a backward model selection procedure and selected the final model with best model fit based on Akaike’s Information Criterion (stepAIC function, MASS library, 67).” (line 349-360). We now slightly rephrased this, and added “… we ran a backward model selection procedure and…” in line 358 Also, we adjusted the caption of table 3 “Final multiple regression model results….” (between line 380-381), to make it more explicit that our results concern multiple regression models.

• Limited explanation of what is influencing the character of floodplain sedimentation is undertaken – the sole focus is on a number of predetermined vegetation variables.

We now address this issue within two new paragraphs in the introduction that the second reviewer suggested (line 141-171). In addition, we added references. The new paragraphs emphasize the abiotic controls of sediment retention on floodplains.

• Some of figures are hard to interpret and require reworking.

We adjusted the figure caption of Fig 1 for more clarity. “Map of the three floodplains in two areas along the Mulde River with trap locations. The grey line is the Mulde River and the black frames are the study areas. Low hydrological distance = Short pathway of lowest elevation the water takes to the plot; High hydrological distance = Long pathway of lowest elevation the water takes to the plot.” (line 208-212)

We improved Fig 2 with colors and text in the figure caption. More links are now made to other tables and figures, which will help the interpretation. “Fig 2. Set-up of different measurements in the vegetation plots (2m x 2m). The set-up of the green sediment traps (image in S2 Figure) and the defined areas of biomass harvest upstream each plot after the flood event. It also shows the set-up for the structural images of the vegetation with the camera 1 m in front and the blue background wall 0.5 m inside the plot (image in S1 Figure).” (line 261-265)

In the figure caption of Fig 3 we specified the biomass. “ … (a) plant biomass, …” (line 381-386).

In the figure caption of Fig 5, we added: “… and grouped by the mean for low and high hydrological distance…”. (line 410-412)

We also changed S1 Figure by including the color image and improving the figure caption. “S1 Figure: Structural image. a) Original image with blue background wall and blue flooring material in front. b) Automatically analysed images for the following predictors as explained in table 1: Vertical density = percent of vegetation pixels on the image of standard size, Mean height = mean height of vegetation pixels on the image, Median height = median height of vegetation pixels on the image, and Height variation = standard deviation of vegetation pixel height on the image.” (line 837-842)

We also re-checked all other figures, but could not find any unclarities ourselves. We would be happy with some more detailed suggestions on which aspects of which figures may benefit from improvement.

• Many of the references used to support significant statements are inappropriate in that they do not represent case studies of similar focus.

Thanks for the comment. We carefully went through the manuscript considering it, improved some wordings and moved a few references as stated in the following:

Removed reference 20 (Elliott 2000) in line 131 and combined the sentences. “Discharge and with it, inundation depth are strongly affected by elevation and with that the location within the floodplain is relevant for sedimentation.”

Removed reference 28 (Akram et al. 2014) in line 133. And added ref 29 (Asselman et al. 1995) in line 134 (27, 29, 40).

We move reference 19 (Gurnell et al. 2012) from line 92 (22,25) to line 77 (18, 19).

However, we would be grateful, if reviewer 1 could specify, if there is any further references she/he thinks are inappropriate to further improve our manuscript revision.

Reviewer #2: GENERAL COMMENTS

This paper presents a very interesting set of spatially-detailed field observations of the aggregate deposition of fine sediments and associated nutrients across a floodplain surface during a relatively low magnitude flood event. The quality of the observations and data analysis are both high and extremely informative. However, I think it is important to place these observations into their hydrogeomorphological context. Only one flood was observed, which is not surprising given the amount of field and laboratory work involved, and there may be many results that can be extracted from that event that are transferable to other events and locations. However:

1. In the later part of the introduction, the authors need to acknowledge the limitations of considering only one flood event. Text along the following lines needs to be inserted somewhere:

“Flood magnitude and duration are the fundamental controls on the delivery of sediment transported by a river from its channel onto its floodplain. Flood magnitude and duration also affect the spatio-temporal distribution of depth and velocity of the inundating water as it flows across the floodplain surface. However, the amount and particle size of sediment retained across the floodplain is strongly moderate by two local, natural, factors. First, floodplain topography imposes strong local variations on the depth and velocity of the flood water. Second, any vegetation cover on the floodplain surface interacts with the flood water, slowing local flow velocities and thus encouraging the settlement of sediment particles from the water. However, because vegetation is flexible, it can respond to high flow velocities by bending and adopting a more streamlined form, and this leads to changes in its ability to intercept and retain sediment as flow depth and velocity vary through time and across space. Understanding of these complex interactions is critical to building a picture of the importance of floodplain topography and vegetation cover for the retention of sediments and associated nutrients. While experiments in laboratory flumes can go a long way towards delivering such understanding, particularly by manipulating flow depth, velocity and transported sediment load across vegetated surfaces displaying different traits, the results of such experiments need to be validated and elaborated within the complexity of real-world situations.

In this paper, we present a set of field observations exploring the detailed influence of natural topographic features and meadow vegetation communities on finer sediment and nutrient retention during a single flood event. Although the results of such field investigations are limited by the magnitude and duration of the investigated event, they can reveal detailed and highly informative spatial patterns that are relevant to floods of similar magnitude at other sites and they can reveal associations that may be applicable to floods of differing magnitude and duration. In the present case, we seek to reveal the detailed impact of topographic setting and different traits of flood meadow vegetation on sediment and nutrient retention during flood events of relatively low magnitude and high frequency.”

We included the whole paragraphs in the introduction (line 141-171).

Accordingly, we also adjusted the first sentence of the next paragraph: “Thus, the aim of this study was to analyse combined effects of vegetation characteristics and selected topographic features at the scale of vegetation patches on the floodplain.“ (line 172-174)

We included a reference that found the importance of “smaller floods” for nutrient retention. “In the present case, we investigated a flood of relatively low magnitude that occur on average biannually, but others already emphasised the importance of low magnitude, but higher frequent floods for nutrient retention on floodplains (46)”. (line 166-169)

2. The magnitude (discharge in m3/s) and frequency (recurrence interval in years) of the investigated flood peak and the likely duration of that flood as an over bank event needs to be more prominent within the description of the study site. At present it is buried in the middle of a paragraph. I would give it a separate paragraph at the end of the Study Site section. I suggest you have three paragraphs – the first is a general context, the second describes the topography and species of the floodplain and the third describes the flood event that was investigated.

We restructured the description of the study site as suggested. First paragraph about the general context (line 193-207), second paragraph about the topography and the species (line 213-218), and third the description of the flood event, where we added another sentence: “However it was the only discharge of such high magnitude that occurred during the 5-years study period (2016-2020)” (line 219-222).

3. The above aspects then need to be acknowledged in the Discussion. In other words, the work has produced some very useful insights that illustrate the importance of natural floodplains for sediment and nutrient retention, but future work needs to look at one or more other floods of different magnitude at the same site to be able to provide a truly integrated perspective on this topic.

We included an outlook section into the discussion, where we suggested to study other floods of the same and of different magnitudes at the same sites (line 546-552).

SPECIFIC COMMENTS

INTRODUCTION

This gives good coverage of the literature, but as stated in my general comments, it needs to recognise the importance of the flood properties. Looking at your list of literature, some (probably many) of those papers have looked at sedimentation over extended periods (aggregating a series of floods) or have separated results from different events, so you can use this literature to support the importance of flood magnitude, duration and sediment load on at-a-site floodplain sedimentation.

Thanks for that comment, we included the above suggested paragraph as it is and added some more references to support the flood properties.

“Flood magnitude and duration are the fundamental controls on the delivery of sediment transported by a river from its channel onto its floodplain (45)” Flood magnitude and duration also affect the spatio-temporal distribution of depth and velocity of the inundating water as it flows across the floodplain surface (46).” (line 141-144)

“First, floodplain topography imposes strong local variations on the depth and velocity of the flood water (20,21,44). Second, any vegetation cover on the floodplain surface interacts with the flood water, slowing local flow velocities and thus encouraging the settlement of sediment particles form the water (20,21,44).” (line 146-149)

“… retain sediment as flow depth and velocity vary through time and across scale (47-49)”. (line 152-153)

In the present case, we investigated a flood of relatively low magnitude that occur on average biannually, but others already emphasised the importance of low magnitude, but higher frequent floods for nutrient retention on floodplain (50)”. (line 166-169)

When you list your hypotheses, you can emphasise the fact that these are investigated within the context of a relatively low magnitude, high frequency event – typical of (for example) the seasonal, annual or biannual events experienced by most river margins in North West Europe

We included a sentence about the specific magnitude and frequency of this flood into the new suggested paragraph in the introduction. It now says: “In the present case, we investigated a flood of relatively low magnitude that occur on average biannually.” (line 166-167).

MATERIALS AND METHODS
Study site: I am not sure of PlosOne regulations, but if it is possible, I would transfer Supplementary Figure 1 into the main manuscript.

We transferred S1 Figure now to the main manuscript. It is now called Fig 1 (line 208-212), thus all other figures (main text and supplementary) changed their numbering accordingly.

I would also restructure this section into three paragraphs as recommended in my general comments (above).

Changed according to the suggestions above. (line 193-222)

Vegetation data and Vegetation characteristics: The work is clearly described but you make no attempt to justify why your observations were in Summer 2016 and Spring 2017 when the flood was in February. Clearly you could not capture this information during the flood, but the difference between the survey and flood dates may have affected the analytical outcomes.

The reason to use both data sets was that both could be relevant to describe the structure of the vegetation. The summer before describes the species and the cover of the vegetation at their biomass peak, but might partly already decomposed during winter before the flood. The data from the “spring 17 after the flood” were collected after the flood has left and tries to represent the vegetation that was present during the flood. We try to include a short explanation into the manuscript. “We did this once in summer 2016 before the flood and once in spring 2017 after the flood, trying to catch the point in time that represents the vegetation structure during the flood best.” (line 245-247) “… to estimate the density and height distribution of the vegetation during the flood.” (line 248-249)

Study design: Once again you have clearly described what you did but not why you did it this way. For example why did you lay out each plot according to the design in Figure 1?

We improve that paragraph about the study design by including more the why, not only the how. It now reads the following:
“Two sediment traps were fixed at the downstream side of each plot, to well represent the conditions of the vegetation on the plot.” (line 276-278).
“In addition, we also harvested the patch of biomass directly in front (up-stream) of the trap, to quantify the vegetation biomass that influence the sedimentation on the trap” (line 287-289).
“In the lab, we quantified the sediment by washing it off the traps…” (line 289-291).

Nutrient analysis: Given the importance of particle size for settlement and also for the ability of the particles to retain nutrients, why did you not conduct a particle size analysis of the trapped sediments? Indeed, an indication of the proportion that was > 2mm or < 2mm would have been interesting and could have supported some of your later discussion of likely particle size differences and influences.

For the nutrient analysis we sieved the sediment < 2 mm (line 297). However, there were rarely sediment larger than 2 mm, therefore we did not include these data. Grain size analysis separating sand (2 - 0.063 mm), silt (0.063-0.002 mm) and clay (< 0.002 mm) would have been the most interesting, however the amount of captured sediment was for many samples not enough to run nutrients and grain size analysis, and technical possibilities were limited for grain size analyses. “Third, increasing the size, length or number of the sediment traps per plot to ensure to capture enough sediment to additionally investigate particle size.” (line 550-552)

Topographical variables: (i) Your calculations of mean water level height are unclear.

We changed the description about the mean water level height, to make it more clear. It now reads the following: “Mean water level height e_mf was calculated using the closest gauging station (Priorau, 560090; daily measurements 1995-2015). The elevation differences between the study areas were derived from the digital elevation model (DEM, © GeoBasis-DE, LVermGeo LSA, [m.E. 2016, C22-7009893-2016]).” (line 309-312).

(ii) I like the idea of the ‘hydrological distance’ but it would have been useful to show that this has greater explanatory power than a simple straight line lateral offset from the bank top, since this latter measure has been used previously.

Thanks for that idea. We rerun the analysis and included the second distance measure “shortest distance from the river edge”. Including both metrics run into collinearity issues. If we compare the model including only the hydrological distance with the model only including the straight line instead shows that the model with the hydrological distance has a higher explanatory power (AIC).

(iii) You need to give a better explanation of why you used longitudinal stream distance because it must be a surrogate for something more meaningful. As you have a DEM, does the gradient of the river channel reduce slightly from upstream to downstream (this would reduce the total energy of the flow and thus the flow velocity) and/or does the channel widen so that it is accommodating the same discharge in a wider channel (this would reduce the river’s energy per unit channel width and again the flow velocity)?

As we explained in the method section: “We included longitudinal stream distances as river kilometre in the study to account for the plot location along the stream, since we visually observed lower flow velocity at the study area further downstream.” (line 321-323)

However, we measured the channel width during the flood peak with the DEM and the gauging station per area. With this rough estimation we can say that the channel of the downstream study area is at least 10 % wider than the channel of the upstream study area. We included this explanation of the findings in relation to the flow velocity into the discussion. “We also know that during the flood peak the channel width of the downstream site was at least 10 % wider than the channel at the upstream site, which causes reduced flow velocity and with that might increases sedimentation. We also visually observed lower flow velocities at the downstream site, at least for those plots close to the river, which might cause higher amounts of fine grained sediment, and thus, higher C and N retention with increasing river kilometre” (line 496-502)

Leaf surface traits: I like your use of a trait data base for leaf area as this removes seasonal effects inherent in any field measurements.

Data analysis: the approach adopted seems fine

RESULTS

Your results are presented clearly and in a logical order.

DISCUSSION

I think you should start by reminding the reader of the limitations of your study: one river, three floodplain sections, one relatively low magnitude flood and 24 plots inundated and thus available for analysis. Having stressed that, you can then move on to the current first paragraph, stressing that within these boundary conditions you have identified high spatial variability in sedimentation quantities and sediment-associated nutrients and have been able to explain a notable proportion of that variability with a range of vegetation and topographic variables. Such explanatory power reveals the more general importance of these local factors in floodplain sedimentation processes.

We clearly address the limitation at the end of the first paragraph of the discussion. “The current study was conducted on three floodplains in one river. The magnitude of the flood was relatively low, but of high frequency (biannual), so that 24 sites were inundated and thus available for the analysis. The explanatory power of the results reveals the more general importance of these factors in floodplain sedimentation processes.” (line 429-432)

Vegetation characteristics:

Point (3) line 385 is not clear to me.

We changed the sentences for clarification: “(3) during the winter decomposition of the plant biomass started and might change the vegetation structure compared to the flume experiment conducted at the biomass peak.” (line 444-446)

Lines 389 to 391 seem rather spurious as you have no particle size data. Maybe you need to rephrase these lines to make it clearer how these findings (by others?) might explain some of your results?

We changed the sentence to clarify the meaning. “Sediment on the vegetation surface is likely to be finer grained (silt and clay), since larger grains might be too heavy to be hold by single leaves.” (line 448-449)

Topography:

One thing that you need to discuss is that your sedimentation rates measured under the plant canopies are actually measured on an artificial vegetation surface! This means that, although this is a standard surface whereby relative differences between plots are comparable, it may not represent actual sedimentation rates on bare earth surfaces. I know this is a problem of all sediment traps, in that they only represent a single simplified surface type, but the issue needs to be mentioned insofar as the absolute values for sediment deposition may be higher or lower than those that would have been obtained by another standardised surface.

We specified it in the paragraph about the ratio of sedimentation on and underneath the traps to clarify that the sedimentation underneath the vegetation is on artificial traps and added a reference (Steiger et al. 2003). “Thus, our results emphasize the crucial role of vegetation for floodplain sedimentation, although the ratio needs to be interpreted with caution, since the sedimentation on the ground underneath the vegetation is represented by artificial traps, even though they are known to best represent ground surface conditions (55).” (line 487-491)

Carbon, nitrogen and phosphorus on the vegetation:

Here again you use particle size as a possible explanation when you have no particle size data. Maybe you should have a section that suggests possible future work that could extend and reinforce your findings?

Thanks for this suggestion. We included an “outlook” section at the end of the discussion before the conclusion, where we also suggested analysis of the particle size. “Third, increasing the size, length or number of sub-samples per plot to ensure to capture enough sediment to additionally investigate particle size.” (line 550-552)

Diversity and leaf surface structure: I have no comments on this section.

As suggested above, I think you could add a final section in the Discussion section that relates to possible extensions of the work. I would suggest: (i) monitoring of a larger flood, which would have the potential to introduce the effects of vegetation deformation (the amount of deformation would vary with the exposure of that vegetation and thus would probably link well with the topography); (ii) the inclusion of a third vegetation-trap strip combination in each plot to ensure that you have enough sediment to investigate particle size as well as nutrients; (iii) possibly an experimental area to compare the impact of using different trap materials to quantify the sedimentation rates below the vegetation?

We added another paragraph in the discussion: the “Outlook” (line 546). Here, we discuss, as suggested, possible extensions of the work. We suggest a monitoring of similar floods of similar magnitude, but we excluded (iii), since this was already done in another study (Steiger et al. 2003, 49), as cited in the method section (line 271-272). The new paragraph now reads the following: “Overall we would suggest some further research to improve our understanding of the mechanisms. First, a monitoring of similar floods of the same magnitude to validate our findings. Second, the monitoring of a larger flood, which would have the potential to introduce the effects of inundation depth and vegetation deformation. Third, increasing the size, length or number of sediment traps per plot to ensure to capture enough sediment to additionally investigate particle size.” (line 547-552).

CONCLUSION

As would be expected, this is a short section. However, after saying what you have shown, you also need to qualify this with some statement about its transferability. I found the statements about management very prescriptive. I would be more cautious but certainly say that because vegetation and topography have important effects on sedimentation, they can also be used to forecast where sedimentation is likely to reach a maximum and also how vegetation might be manipulated to deliberately enhance sedimentation in certain areas.

We worked on the conclusion, and included a statement about its transferability at the end of the paragraph. “ Overall, our findings are transferable in a mechanistic perspective. We are not able to predict any quantitative sedimentation for other floodplains, flood or river catchments, but the mechanistic approach allows to tackle the key characteristics that in general cause the process of sedimentation on floodplain meadows.” (line 579-583)

We also tried to clarify that the management strategies are not prescriptive rather suggestions and recommendations. “First, reduced mowing for more standing vegetation biomass during the flood season would increase sediment and nutrient retention via the total biomass.” (line 564-565) “The present study shows, that the mowing regime…” (line 567).

In addition, we referenced two recent studies that emphasized the importance of lateral connectivity of floodplains for nutrient retention. “…including long abandoned meanders and depressions, which also has recently been found for nutrient retention in other studies (82,83).” (line 574-575)
